# Extensive Chromosomal Reorganization in *Apistogramma* Fishes (Cichlidae, Cichlinae) Fits the Complex Evolutionary Diversification of the Genus

**DOI:** 10.3390/ijms20174077

**Published:** 2019-08-21

**Authors:** Gideão Wagner Werneck Félix da Costa, Marcelo de Bello Cioffi, Thomas Liehr, Eliana Feldberg, Luiz Antonio Carlos Bertollo, Wagner Franco Molina

**Affiliations:** 1 Departamento de Biologia Celular e Genética, Centro de Biociências, Universidade Federal do Rio Grande do Norte, Natal 59078-970, RN, Brazil; 2 Departamento de Genética e Evolução, Universidade Federal de São Carlos (UFSCar), Rodovia Washington Luiz, Km. 235, C.P. 676, São Carlos 13565-905, SP, Brazil; 3 Institute of Human Genetics, Friedrich Schiller University, Am Klinikum 1, 07747 Jena, Germany; 4 Instituto Nacional de Pesquisas da Amazônia, Laboratório de Genética Animal, Av. André Araújo, 2936, Manaus 69077-000, AM, Brazil

**Keywords:** Neotropical cichlids, dwarf species, repetitive DNAs, karyotype evolution, species flock, postzygotic isolation

## Abstract

Neotropical cichlid fishes are one of the most diversified and evolutionarily successful species assemblages. Extremely similar forms and intraspecific polychromatism present challenges for the taxonomy of some of these groups. Several species complexes have a largely unknown origin and unresolved evolutionary processes. Dwarf cichlids of the genus *Apistogramma*, comprising more than a hundred species, exhibit intricate taxonomic and biogeographic patterns, with both allopatric and sympatric distributions. However, karyotype evolution and the role of chromosomal changes in *Apistogramma* are still unknown. In the present study, nine South American *Apistogramma* species were analyzed using conventional cytogenetic methods and the mapping of repetitive DNA sequences [18S rDNA, 5S rDNA, and (TTAGGG)n] by fluorescence in situ hybridization (FISH). Our results showed that *Apistogramma* has unique cytogenetic characteristics in relation to closely related groups, such as a reduced 2n and a large number of bi-armed chromosomes. Interspecific patterns revealed a scenario of remarkable karyotypic changes, including a reduction of 2n, the occurrence of B-chromosomes and evolutionary dynamic of rDNA tandem repeats. In addition to the well-known pre-zygotic reproductive isolation, the karyotype reorganization in the genus suggests that chromosomal changes could act as postzygotic barriers in areas where *Apistogramma* congeners overlap.

## 1. Introduction

Cichlid fishes, representing one of the most impressive examples of adaptive radiation, are currently distributed in tropical America, Africa and Madagascar, and South Asia [1]. Of approximately 1700 species, 571 occur in the Neotropical region [2], where they constitute one of the most diversified groups [3].

Although factors that promoted the exceptional diversity of some Neotropical cichlid clades, represented by the subfamily Cichlinae, are still not completely known, dispersal and vicariance events [4,5], as well as the distinct ecological opportunities and rich species behavioral repertory [6], have been suggested as triggers for their biological diversification.

The biological diversification of Geophagini, the most diversified Cichlinae tribe with more than 300 species [7,8], is coupled with cryptic diversity, generally associated with complex chromosome evolution [9,10,11,12,13,14]. In this clade, species with significant diversity and small body size, the so-called “dwarf cichlids” [15], including the genus *Apistogramma* Regan (1913), are yet largely unexplored with regard to their chromosomes.

Members of *Apistogramma*, an extremely species-rich group, with 112 valid species [16] and a large number of species yet to be formally described [17], have a wide distribution throughout most of South America, specifically in the eastern part of the Andes [4]. Although distributed over vast areas, the species are generally restricted to small watersheds or endemic to a single river (e.g., [18,19,20]). *Apistogramma* species represent the smallest cichlids known, ranging from 2 to 8 cm in size [21,22], with a marked sexual dimorphism, where males are larger and exhibit more exuberant color patterns [18].

Due to its high endemism, sympatry levels, and species diversity, *Apistogramma* has several provisional taxonomic groups, whose phylogenetic relationships are largely uncertain [4,23]. Its morphological taxonomy is particularly difficult, based on many cases in cryptic differences and body color patterns [4,17]. Up to now, phylogenetic inferences based on nuclear and mitochondrial markers have identified four *Apistogramma* clades in three main morphological groups, namely the *steindachneri*, *agassizii*, and *regani* lineages [23], reinforcing the difficulties in using the morphospecies concept to establish evolutionary relationships.

Given the wide diversity and geographic overlap of *Apistogramma* species, cytogenetic analyses can help in understanding the evolutionary processes and present novel explanations for species integrity in sympatry scenarios. In fact, chromosomal rearrangements fixed in allo/parapatry can constitute more effective interspecific genetic barriers than those dependent exclusively on the accumulated changes in allele sets [24]. Accordingly, alterations in numbers and structure of chromosomes may abruptly change linkage groups, thus reducing recombination [25,26] and thereby protecting genomic regions from introgression and favoring speciation [27].

The diversity of *Apistogramma* species, taxonomic complexity, vast sympatry level, and the extreme anthropic vulnerability of some species [28,29] reinforce the need to understand their biological diversity, species limits and speciation processes. In this scenario, cytogenetic data may provide useful information for approaches to study their biodiversity [30]. Nevertheless, until now, the available cytogenetic characteristics are still incipient for *Apistogramma*, generally restricted to conventional karyology [31,32]. To fill this gap, here we present a detailed survey of chromosomal diversification and genomic organization in species of this genus. To ensure broad phylogenetic representation, nine representatives of different species complexes were analyzed using conventional (C-banding, Ag-NORs) and molecular methods, by fluorescence in situ hybridization (FISH) with 5S rDNA, 18S rDNA, and telomeric (TTAGGG)_n_ probes. The obtained extensive chromosomal data showed a remarkable karyotype macrostructure diversification, indicating that chromosomal changes have played an important role in the evolutionary divergence of this exuberant Neotropical cichlid group.

## 2. Results

**Figure 1:**
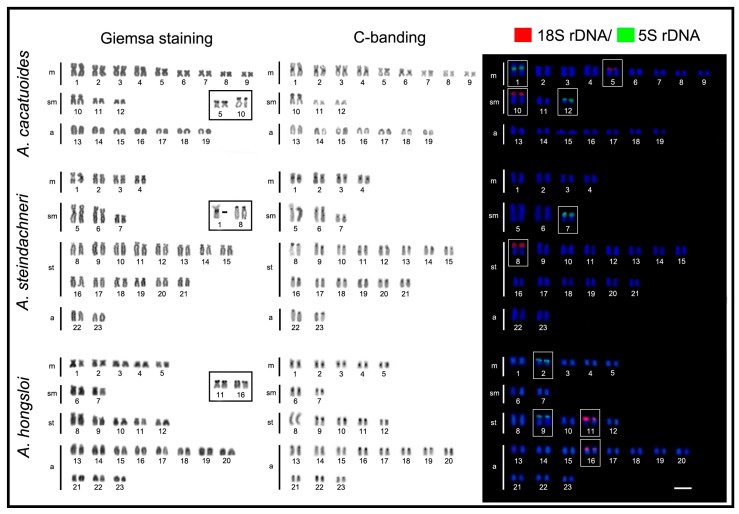
Karyotypes of *Apistogramma cacatuoides*, *Apistogramma steindachneri*, and *Apistogramma hongsloi*, arranged after Giemsa-stained, C-banding, Ag-NOR, and fluorescence in situ hybridization (FISH) with 18S rDNA (red) and 5S rDNA (green) probes. The Ag-NOR-, 18S-, and 5S-bearing chromosomes are boxed. Bar = 5 μm.

**Figure 2:**
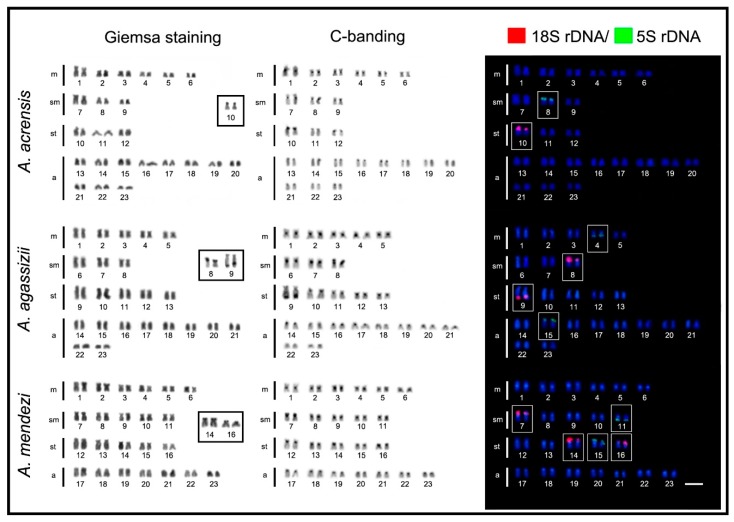
Karyotypes of *Apistogramma acrensis, Apistogramma agassizii*, and *Apistogramma mendezi* species arranged as described in Figure 1.

**Figure 3:**
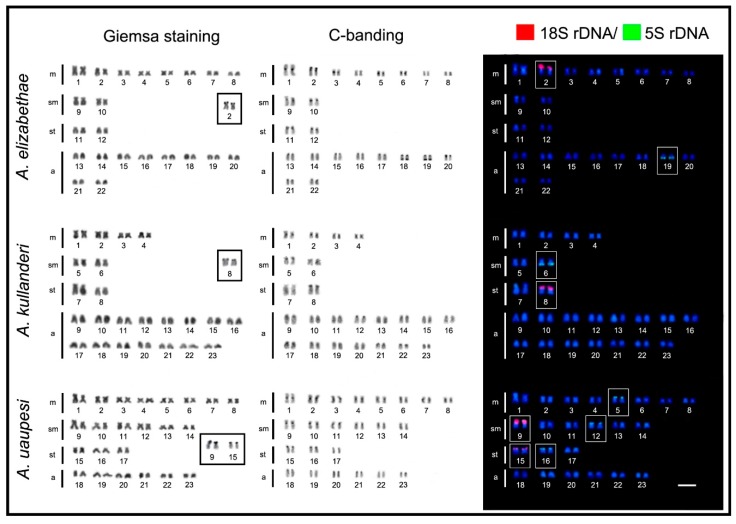
Karyotypes of *Apistogramma elizabethae*, *Apistogramma kullanderi*, and *Apistogramma uaupesi* arranged as described in Figure 1.

**Figure 4:**
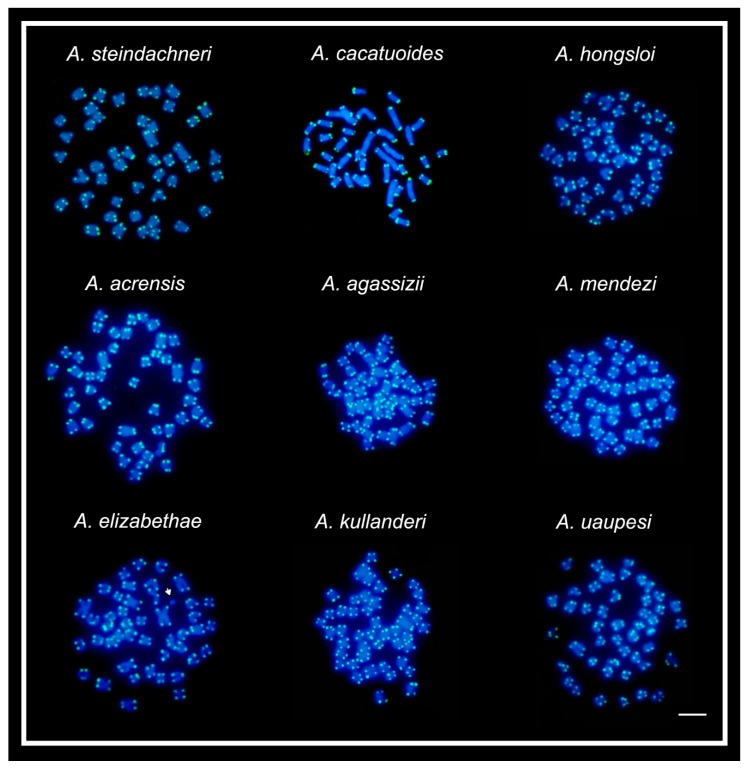
In situ hybridization with telomeric probe (TTAGGG)_n_ in somatic metaphases of nine *Apistogramma* species. The arrow shows B-chromosome in *Apistogramma elizabethae* complement without hybridization signals. Bar = 5 μm.

All *Apistogramma* species exhibited 2n = 46 chromosomes, except *A. cacatuoides* and *A. elizabethae*, which had 2n = 38 and 2n = 44 chromosomes, respectively (Figure 1, Figure 2 and Figure 3). In the latter species, a small B-chromosome was found in 30% of metaphases of one individual (Figure 4). In general, the species showed diversified karyotypes, composed mainly of biarmed chromosomes. The number of chromosome arms (NF) values ranged from 56 to 72, with 62 being the most frequent one (see Table 1).

The C-positive heterochromatic bands were primarily located in the centromeric regions, with additional and most conspicuous heterochromatic blocks coinciding with the NORs in all species (Figure 1, Figure 2 and Figure 3). The Ag-NOR sites were located in one, two, or three chromosome pairs (Figure 1, Figure 2 and Figure 3; Table 1), with a preferential location in the terminal region of the short arms (Figure 1, Figure 2 and Figure 3). The mapping of the 18S rDNA sites was, in general, consistent with Ag-NOR staining (Figure 1, Figure 2 and Figure 3; Table 1), except in *A. steindachneri*, which exhibited extraribosomal argentophilic sites (Figure 1), and *A. mendezi,* with one 18S rDNA site without a corresponding Ag-NOR signal (Figure 1).

The 5S rDNA sites showed a diversified occurrence in one, two, or three chromosome pairs among species, but with a non-syntenic location with the 18S rDNA sites (Figure 3). In all species, both rDNA classes displayed a preferential localization in the terminal region of the chromosomes, except in *A. cacatuoides*, where they occupied the pericentromeric position of the largest m pair (Figure 1). The mapping of telomeric sequences (TTAGGG)_n_ revealed exclusive hybridization signals at the terminal positions of the chromosomes, as expected, with no visible additional interstitial telomeric sites (ITSs) (Figure 4).

## 3. Discussion

### 3.1. Chromosomal Evolutionary Pathways in the Apistogramma genus

The impressive evolutionary diversification of cichlids has been associated with karyotype changes that usually reflect the large-scale biogeographic patterns of their lineages. The African clades exhibit a variation of the 2n number from 32 to 48, and karyotypes with predominantly 44 chromosomes. In contrast, the South American clades (Cichlinae) present a wide range of variation, from 2n = 38 to 60, and a large predominance of 48 chromosomes, primarily of st/a categories [9,11].

The karyotype evolution of Neotropical cichlids has revealed trends of conservation, reduction or increase of the diploid number. Thus, (i) while some groups share 2n = 48, with variations caused by pericentric inversions or other types of centromeric shifts (including non-reciprocal translocations), (ii) others show a reduction of this value (2n < 48) due to chromosome fusions; and finally, (iii) some others present karyotypes with a high 2n value (50 to 60), mainly encompassing centric fissions and reciprocal translocations events (e.g., [9,33]). However, such general trends may exhibit independent evolutionary pathways among specific groups of a tribe.

**Figure 5:**
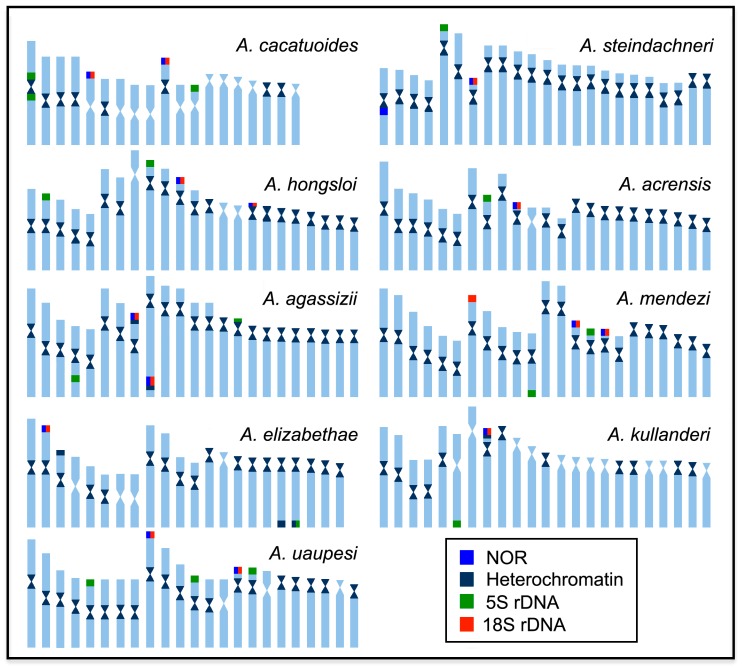
Idiograms detailing the karyotype structure and organization of rDNA sequences and heterochromatin in *Apistogramma* species.

In general, the chromosomal patterns of the speciose tribe Geophagini reveal largely conserved karyotypes, with 2n = 48 formed by st/a chromosomes [11,34], in which only some pericentric inversions may be detected [9]. However, among the Apistogrammines (*Apistogramma + Taeniacara + Satanoperca*) (*sensu* [7]), the genus *Apistogramma* exhibits an exclusive karyotype macrostructure pattern within its own clade and in the Geophagini tribe. In this group, the reduced karyotypes (2n ≤ 46) with a high number of bi-armed chromosomes (Figure 5) largely contrast with those found in the other Apistogrammines (2n = 48). Such a low 2n value originated from one ancestral fusion event, at the onset of the diversification of the genus by around 55 Mya [23].

**Figure 6:**
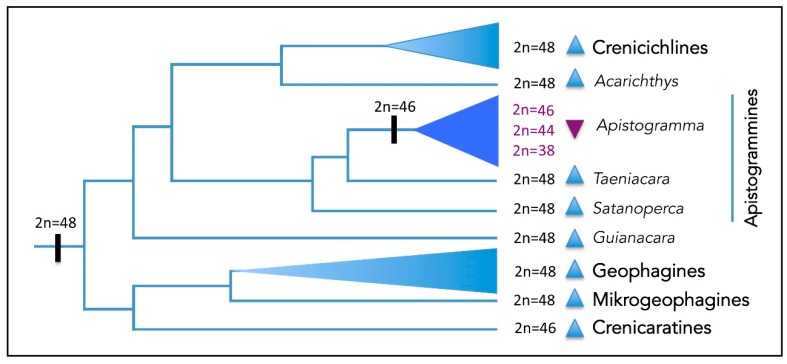
Diploid numbers (2n) and karyotype structure of Geophagini groups under a phylogenetic perspective [7]. Blue triangles represent karyotypes with >50% acrocentric chromosomes shared by several Cichlinae clades and some Apistogrammines groups; the purple triangle shows the exclusive diploid set found for the genus *Apistogramma*, characterized by a reduced chromosome number with <50% acrocentric chromosomes.

The set of particular karyotype traits in *Apistogramma* provides a conspicuous phylogenetic signature, which supports the monophyly of the genus (Figure 6), as also supported by morphological and molecular data [4,23]. In addition to this basal fusion event, characteristic of the *Apistogramma* genus, a multitude of secondary and stochastic chromosomal changes, caused by pericentric inversions (or non-reciprocal translocations) and Robertsonian translocations, promoted additional numerical and structural karyotype changes. In fact, these changes are visible through the extensive NF variation among species (NF = 54–72), and 2n reductions in *A. elizabethae* (2n = 44) and *A. cacatuoides* (2n = 38).

### 3.2. The Complex Chromosomal Diversification in Apistogramma Species Flock

*Apistogramma* represents a fascinating example of fish karyotype diversification in continental environments. Ten out of 12 species already analyzed display karyotypes with 2n = 46 chromosomes, predominantly bi-armed ones (Table 1). Notably, the karyotypes differ among each other by structural rearrangements, especially pericentric inversions/non-reciprocal translocations but also, although in a lesser extent, numerical–structural changes (centric fusions) pointing to the trend of 2n reduction in the genus.

*Apistogramma* encompasses various species complexes characterized by cryptic or inconspicuous distinction among its species, which might be frequently associated with incipient speciation. This pattern, typically found in the African cichlids (e.g., [35]), has also been observed in some genera of Neotropical cichlids, in which suspected cryptic species have been identified based on morphological, genetic, and cytogenetic approaches [36,37]. In species complexes, the rate of karyotype differentiation is variable, ranging from conservative, regarding the structure and internal organization [38], to highly divergent (e.g., [39]).

Species complexes may constitute a species flock, i.e., groups of closely related species with the significant congruence of diversity and spatial distribution [40,41]. *Apistogramma* fulfills these conditions in many areas of its distribution, in which a high level of endemism and species diversity occur. This condition stands out in the Western Amazon, particularly in Peru (21 endemic species), Orinoco River (19 endemic species), and Negro River basins (13 endemic species) [42]. Its species have a very extensive geographic distribution in several South American watersheds, the reflex of a complex biogeographic history [4], showing a temporal sequence of cladogenetic events associated with dramatic chromosomal changes. This condition contrasts with the one found in Tilapinii cichlids (African lineages), where more than a hundred species display highly conserved karyotypes [11].

It is likely that the surprising diversification of *Apistogramma* accompanied by broad chromosomal differentiation indicates that chromosome rearrangements may have had active participation in the cladogenesis of the group. A similar relation, to a lesser degree, has been reported for a number of other species complexes of Neotropical cichlids [9,34].

Chromosome reorganizations in several fish groups have been associated with complex sequences of repetitive DNA [43,44,45,46]. However, in the *Apistogramma* species, the heterochromatic regions exhibited conservative pericentromeric patterns, not evidencing provisionally an organization particularly diverse.

Among the Cichlinae fishes, the occurrence of NORs in a single chromosome pair, with variations in location between the long and short arms, represents the most frequent and basal condition [9,47,48]. However, non-rare rDNA sites are variable, with the presence of multiple Ag-NOR/18S rDNA sites [13,33,49].

**Figure 7:**
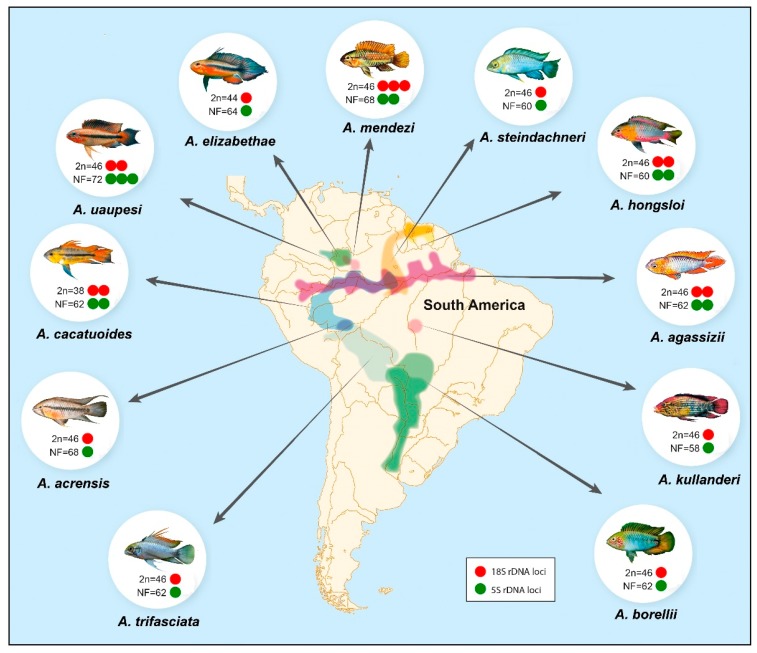
Diagrammatic representation of the main cytogenetic features of the analyzed *Apistogramma* species and pertaining areas of occurrence in South America. Cytogenetic data and areas of occurrence of species are indicated by colors.

The *Apistogramma* species showed a very diversified distribution and frequency of rDNA regions (Figure 5 and Figure 7), in contrast to other South American species complexes, like *Crenicichla* [18,36,50], which have a single Ag-NOR-bearing chromosome pair [34]. In fact, they can have one, two, or three chromosome pairs bearing Ag-NOR/18S rDNA sites.

Among its species, some incongruence between 18S rDNA sites and Ag-NOR signals was observed in *A. steindachneri* (additional Ag + site) and *A. mendezi* (additional 18S rDNA loci). The first case could be associated with pseudo-NORs [51,52], and the second case possibly indicates the occurrence of pseudogenes [53] or epigenetically silenced regions [54].

In *Satanoperca* species, a group phylogenetically close to *Apistogramma* Ag-NOR sites can be single [11,55] and also located in two chromosome pairs [56,57], indicating that the diversification of the rDNA clusters in *Apistogramma* is ancestrally shared with other Apistogrammines species.

Dual-color FISH with 5S and 18S rDNA showed their non-syntenic localization in *Apistogramma* chromosomes, the most common arrangement in fishes [11,13,48]. However, 18S and 5S rDNA sites vary in number and location in the chromosomes of species. In *A. cacatuoides*, 5S rDNA sites are located in the pericentromeric region of the largest metacentric pair, suggesting their association with centric fusion rearrangements, as also related in other fish species [38,58,59,60]. As a whole, the mapping of rDNA sequences revealed a dynamic diversification of these repetitive sequences supporting a high reorganization level of the chromosomes of the *Apistogramma* species.

In contrast to other species, *A. elizabethae* harbors a small B chromosome in one individual. B chromosomes usually arise from the normal complement of the species [61] and have been documented for more than 20 South American cichlid species, in which micro or small chromosomes are predominant [62]. In *A. elizabethae,* its occurrence is likely associated with the numerical and structural chromosome changes, including asynchronous fusion rearrangements. Despite its evident occurrence, the chromosome fusion processes in *A. elizabethae* and other species have no preserved interstitial telomeric sequences (ITSs), like it occurs in some other fish species [38,45].

### 3.3. Evolutionary Divergence in the Apistogramma genus. A General View

Multiple lines of evidence indicate that several *Apistogramma* complexes underwent rapid speciation processes or are undergoing a dynamic process of diversification [23]. In the incipient speciation context, the genetic cohesion of groups is insured by prezygotic reproductive barriers [63]. In fact, prezygotic reproductive isolation, where body color plays a decisive role in mate choice, is an evolutionary driving force favoring the sympatric occurrence of a considerable number of *Apistogramma* species [28,63,64]. However, prezygotic mechanisms based on recognition patterns may eventually get abolished by environmental changes or interaction among species [65], as indicated by numerous *Apistogramma* hybrids obtained in captivity, even among little-related species (available online: www.apistogramma.com).

The acquisition of effective postzygotic barriers, which represent the last stage of isolation of specific gene pools, may occur very slowly in some groups. In birds, viable hybrids can occur in species with 55 Myr of divergence [66]. In fish, postzygotic reproductive isolation is estimated to be 11.6 Myr [67], a period considerably longer than the emergence of many current *Apistogramma* species that underwent diversification during the Miocene and glacial cycles in the Quaternary [23].

*Apistogramma* provides a surprisingly dynamic panel of karyotype diversification and its role in the phyletic divergence. The evolutionary diversification and biogeographic patterns in this genus were complex, encompassing numerous small or large vicariant events [23]. The resulting cladogenetic events are clearly related to its karyotype evolution. The chaotic pattern of chromosomal changes during the myriad of allopatric events and historical recontacts likely play an important evolutionary role as a postzygotic barrier, preventing genetic introgressions in locations with a high level of sympatric species.

## 4. Materials and Methods

### 4.1. Individuals, Chromosomal Preparations, DNA Extraction

Nine species of the genus *Apistogramma* from different localities in South America were obtained from ornamental trade and used in cytogenetic analyses (Table 2).

Individuals were submitted to mitotic stimulation with attenuated antigen complexes for 24 h, according to Molina et al. [68]. Next, the animals were euthanized with an overdose of clove oil (Eugenol). Chromosome preparations were obtained from short-term kidney cell cultures [69], and the sex was identified by microscopic examination of the gonads. The experiments followed ethical rules approved (2/9/2015) by the Animal Ethics Committee of the Federal University of Rio Grande do Norte (Process #44/2015).

Total genomic DNA (50–100 ng/μL) was extracted from muscle and liver fragments [70] and stored in 100% ethanol at −20 °C.

### 4.2. Conventional Chromosome Staining

The nucleolus organizer regions (NORs) and distribution of constitutive heterochromatin were analyzed using the methodologies of Howell and Black [71] and Sumner [72], respectively.

### 4.3. Fluorescence In Situ Hybridization

The FISH protocol was performed according to Pinkel et al. [73]. The 5S and 18S rDNA sequences were detected simultaneously by dual-color FISH. The 5S rDNA (200 bp) and 18S rDNA (1400 bp) probes were obtained by PCR, from the nuclear DNA of *Apistogramma agassizii*, using the primers A 5′-TAC GCC CGA TCT CGT CCG ATC-3′ and B 5′-CAG GCT GGT ATG GCC GTA AGC-3′ [74], and NS1 5′-GTA GTC ATA TGC TTG TCT C-3′ and NS8 5′-TCC GCA GGT TCA CCT ACG GA-3′ [75], respectively. The 5S probes were labeled with biotin-14-dATP and the 18S with digoxigenin-11-dUTP, using the nick translation kit according to the manufacturer’s recommendations (Roche, Mannheim, Germany). The slides were treated with DNAse free RNAse A (20 mg/mL in 2xSSC) for 1 h at 37 °C, digested in HCl 10 mM containing 500 μg/mL pepsin for 10 min at 37 °C, fixed with 1% formaldehyde for 10 min, and then dehydrated in an alcohol series. The spreads were incubated in 70% formamide/2xSSC at 72 °C for 5 min. The hybridization solution, consisting of 50% formamide, 2xSSC, 10% dextran sulfate, and the denatured probe (5 ng/µL), in a final volume of 30 μL, was dropped onto the slides and hybridization was performed for 14 h at 37 °C in a moist chamber containing 2xSSC. Two post-hybridization washes were carried out on a shaker (150 rpm) at 37 °C; the first one in 50% formamide 2xSSC for 15 min, the second one in 2xSSC for 15 min. A final wash was performed at room temperature in 4xSSC for 15 min. Avidin-FITC (Vector, Burlingame, CA, USA) was used for signal detection of the 5S rDNA probe and anti-digoxigenin rhodamine (Vector, Burlingame, CA, USA) for 18S rDNA probe. The chromosomes were counterstained and mounted with Vectashield/DAPI (1.5 µg/mL) (Vector, Burlingame, CA, USA).

Hybridization with telomeric probes (TTAGGG)_n_ was carried out using the Telomere PNA FISH kit (Dako Cytomation, Hamburg, Germany), according to the manufacturer’s instructions.

### 4.4. Karyotype Analysis

At least 30 metaphases of each individual were analyzed to characterize 2n and karyotype macrostructure. Mitotic metaphases were photographed with an Olympus BX51 epifluorescence microscope, coupled with an Olympus DP72 digital image capture system, using cellSens Standard 1.7 software (Olympus Corporation, Ishikawa, Japan). Chromosomes were classified based on their arm ratios, according to Levan et al. [76], as metacentric (m), submetacentric (sm), subtelocentric (st), and acrocentric (a) and arranged in decreasing order of size. The definition of NF (number of chromosome arms) considered st-a chromosomes and m-sm ones as having one and two arms, respectively.

## 5. Conclusions

The genus *Apistogramma* offers a classic view of the evolutionary driving forces acting in biological diversification. Its high morphological, chromatic, and cytogenetic plasticity reflects the evolutionary factors acting in small populations, promoting rapid changes in the gene pool. The phylogenetic relationships of species in the genus, based on morphological and molecular data, are contradictory and have not been fully elucidated. Our cytogenetic analysis revealed a remarkable karyotype diversification compatible with the dynamic evolutionary history of this group. The main changes in chromosomes are pericentric inversions (or non-reciprocal translocations) associated with Robertsonian translocations, resulting in complete karyotype differentiation among the species. In addition, the variation of the 18S and 5S rDNA sites supports internal chromosome reorganization. Although prezygotic reproductive isolation has a well-known role in the *Apistogramma* diversification, the intense karyotype rearrangements identified here strongly suggest its participation as a postzygotic barrier contributing to the astonishing diversification of this dwarf cichlid group.

## Figures and Tables

**Table 1:** Cytogenetic data of *Apistogramma* species.

Species	2n	Karyotype Formula	NF	18S rDNA	5S rDNA	Ref.
*A. acrensis*	46	12m + 4sm + 6st + 24a	62	Unique (st-T)	Unique (sm-T)	*
*A. agassizii*	46	12m + 4sm + 8st + 22a	62	Multiple—2 pairs (sm/st-T)	Multiple—2 pairs (m/a-T)	*
*A. agassizii*	46	24m/sm + 22st/a	70	-	-	[31]
*A. borellii*	46	16m/sm + 30st/a	62	-	-	[11]
*A. cacatuoides*	38	18m + 6sm + 14a	62	Multiple—2 pairs (m/sm-T)	Multiple—2 pairs (m-I, st-T)	*
*A. elizabethae*	44	16m + 4sm + 4st + 20a	64	Unique (m-T)	Unique—(a-T)	*
*A. hongsloi*	46	10m + 4sm + 10st + 22a	60	Multiple—2 pairs (m/sm-T)	Multiple—2 pairs (st/a-T)	*
*A. kullanderi*	46	8m + 4sm + 4st + 30a	58	Unique (st-T)	Unique (sm-T)	*
*A. mendezi*	46	12m + 10sm + 10st + 14a	68	Multiple—3 pairs (1sm/2a-T)	Multiple—2 pairs (m/sm-T)	*
*A. ortmanni*	46	24m/sm + 22st/a	70	-	-	[31]
*A. steindachneri*	46	8m + 6sm + 28st + 4a	60	Unique (st-T)	Unique (sm-T)	*
*A. trifasciata*	46	16m/sm + 30st/a	62	-	-	[32]
*A. uaupesi*	46	18m + 8sm + 6st + 14a	72	Multiple—2 pairs (sm/st-T)	Multiple—3 pairs (m/sm/st-T)	*

* present paper; NF—number of chromosome arms; metacentric/submetacentric, two-arm chromosome; subtelocentric/acrocentric, one-arm chromosome. I—interstitial position; T—terminal position.

**Table 2:** *Apistogramma* species analyzed with the extent of geographic distributions.

Species Complex	Species	Geographic Distribution	N	Sex
*A. cacatuoides*	*A. cacatuoides* Hoedeman, 1951	E	6	3 ♂, 2 ♀, 1 immature
*A. steindachneri*	*A. steindachneri* Regan, 1908	E	5	2 ♂, 3 ♀
*A. agassizii*	*A. agassizii* (Steindachner, 1875)	E	3	2 ♂, 1 ♀
*A. agassizii*	*A. elizabethae* Kullander, 1980	R	2	2 ♂
*A. agassizii*	*A. mendezi* Römer, 1994	R	2	1 ♂, 1 ♀
*A. regani?*	*A. kullanderi* Varella and Sabaj Pérez, 2014	VR	1	1 ♀
*A. regani*	*A. acrensis* Staeck, 2003	R	3	2 ♂, 1 ♀
*A. pertensis*	*A. uaupesi* Kullander, 1980	R	2	2 ♂
*A. diplotaenia*	*A. diplotaenia*	R	3	2 ♂, 1 ♀
*A. macmasteri*	*A. hongsloi*	R	2	2 ♀

N—number of individuals analyzed; Geographic distribution: E—extensive; R—restricted; VR—very restricted.

## References

[1] Kocher T.D. (2004). Adaptive evolution and explosive speciation: The cichlid fish model. Nat. Rev. Genet..

[2] Eschmeyer W.N., Fricke R., van der Laan R. Catalog of Fishes: Genera, Species, References. http://www.calacademy.org/scientists/projects/catalog-of-fishes.

[3] Reis R.E., Albert J.S., Di Dario F., Mincarone M.M.M., Petry P.L., Rocha L.A.R. (2016). Fish biodiversity and conservation in South America. J. Fish Biol..

[4] Römer U. (2006). Cichlid Atlas: Natural History of South American Dwarf Cichlids.

[5] Turner G.F. (2007). Adaptive radiation of cichlid fish. Curr. Biol..

[6] Arbour J.H., López-Fernández H. (2016). Continental cichlid radiations: Functional diversity reveals the role of changing ecological opportunity in the Neotropics. Proc. R. Soc. B Biol. Sci..

[7] López-Fernández H., Winemiller K.O., Honeycutt R.L. (2010). Multilocus phylogeny and rapid radiations in Neotropical cichlid fishes (Perciformes: Cichlidae: Cichlinae). Mol. Phylogenet. Evol..

[8] López-Fernández H., Arbour J.H., Winemiller K.O., Honeycutt R.L. (2013). Testing for ancient adaptive radiations in neotropical cichlid fishes. Evolution.

[9] Feldberg E., Porto J.I.R., Bertollo L.A.C., Val A.L., Kapoor B.G. (2003). Chromosomal changes and adaptation of cichlid fishes during evolution. Fish Adaptation.

[10] Ferreira I.A., Poletto A.B., Kocher T.D., Mota-Velasco J.C., Penman D.J., Martins C. (2010). Chromosome evolution in African cichlid fish: Contributions from the physical mapping of repeated DNAs. Cytogenet. Genome Res..

[11] Poletto A.B., Ferreira I.A., Cabral-de-Mello D.C., Nakajima R.T., Mazzuchelli J., Ribeiro H.B., Venere P.C., Nirchio M., Kocher T.D., Martins C. (2010). Chromosome differentiation patterns during cichlid fish evolution. BMC Genet..

[12] Valente G.T., Mazzuchelli J., Ferreira I.A., Poletto A.B., Fantinatti B.E.A., Martins C. (2011). Cytogenetic mapping of the retroelements Rex1, Rex3 and Rex6 among cichlid fish: New Insights on the chromosomal distribution of transposable elements. Cytogenet. Genome Res..

[13] Schneider C.H., Gross M.C., Terencio M.L., Artoni R.F., Vicari M.R., Martins C., Feldberg E. (2013). Chromosomal evolution of neotropical cichlids: The role of repetitive DNA sequences in the organization and structure of karyotype. Rev. Fish Biol. Fish..

[14] Hodanova L., Kalous L., Musilova Z. (2014). Comparative cytogenetics of Neotropical cichlid fishes (Nannacara, Ivanacara and Cleithracara) indicates evolutionary reduction of diploid chromosome numbers. Comp. Cytogenet..

[15] Astudillo-Clavijo V., Arbour J.H., López-Fernández H. (2015). Selection towards different adaptive optima drove the early diversification of locomotor phenotypes in the radiation of Neotropical geophagine cichlids. BMC Evol. Biol..

[16] Fricke R., Eschmeyer W.N., Fong J.D. Species by Family/Subfamily. http://researcharchive.calacademy.org/research/ichthyology/catalog.

[17] Staeck W., Schindler I. (2016). *Apistogramma sororcula*, a new dwarf cichlid. (Teleostei: Cichlidae) from the drainage of the rio. Guaporé in Bolivia and Brazil. Vertebr. Zool..

[18] Kullander S.O., Reis R.E., Kullander S.O., Ferraris J.C.J. (2003). Check List of the Freshwater Fishes of South and Central America.

[19] Britzke R., Mehanna M. (2010). Status taxonômico de *Apistogramma* Regan, 1911 e sua classificação. Bol. Da Soc. Bras. De Ictiol..

[20] Mesa S.L.M., Lasso C.A. (2011). Revisión del género *Apistogramma* Regan 1913 (Perciformes, Cichlidae) en la cuenca del río Orinoco. Serie Editorial Recursos Hidrobiológicos y Pesqueros Continentales de Colombia.

[21] Varella H.R., Sabaj Pérez M.H. (2014). A titan among dwarfs–*Apistogramma kullanderi*, new species (Teleostei: Cichlidae). Ichthyol. Explor. Freshw..

[22] Steele S.E., López-Fernández H. (2014). Body size diversity and frequency distributions of Neotropical cichlid fishes (Cichliformes: Cichlidae: Cichlinae). PLoS ONE.

[23] Tougard C., García Dávila C.R., Römer U., Duponchelle F., Cerqueira F., Paradis E., Guinand B., Chávez C.A., Salas V., Quérouil S. (2017). Tempo and rates of diversification in the South American cichlid genus *Apistogramma* (Teleostei: Perciformes: Cichlidae). PLoS ONE.

[24] Navarro A., Barton N.H. (2003). Accumulating postzygotic isolation genes in parapatry: A new twist on chromosomal speciation. Evolution.

[25] Brown J.D., O’Neill R.J. (2010). Chromosomes, conflict, and epigenetics: Chromosomal speciation revisited. Annu. Rev. Genom. Hum. Genet..

[26] Lukhtanov V.A., Dincă V., Talavera G., Vila R. (2011). Unprecedented within-species chromosome number cline in the Wood White butterfly *Leptidea sinapis* and its significance for karyotype evolution and speciation. BMC Evol. Biol..

[27] Hoffmann A.A., Rieseberg L.H. (2008). Revisiting the impact of inversions in evolution: From population genetic markers to drivers of adaptive shifts and speciation?. Annu. Rev. Ecol. Evol. Syst..

[28] Römer U., Engelking B., Beisenherz W. (2014). Genetically determined mate choice can be influenced by learning in *Apistogramma cacatuoides* Hoedeman, 1951 (Teleostei, Cichlidae). Vertebr. Zool..

[29] IUCN. https://www.iucnredlist.org.

[30] Cioffi M.B., Molina W.F., Artoni R.F., Bertollo L.A.C., Tirunilai P. (2012). Chromosomes as tools for discovering biodiversity–the case of Erythrinidae fish family. Recent Trends in Cytogenetic Studies–Methodologies and Applications.

[31] Thompson K.W. (1979). Cytotaxonomy of 41 species of Neotropical Cichlidae. Copeia.

[32] Roncati H.A., Pastori M.C., Fenocchio A.S. (2007). Cytogenetic studies and evolutive considerations on fishes of the family Cichlidae (Perciformes) from Paraná River (Argentina). Cytologia.

[33] Gross M.C., Schneider C.H., Valente G.T., Martins C., Feldberg E. (2010). Variability of 18S rDNA locus among Symphysodon fishes: Chromosomal rearrangements. J. Fish Biol..

[34] Benzaquem D.C., Feldberg E., Porto J.I.R., Gross M.C., Zuanon J.A.S. (2008). Cytotaxonomy and karyoevolution of the genus *Crenicichla* (Perciformes, Cichlidae). Genet. Mol. Biol..

[35] Salzburger W., Meyer A., Baric S., Verheyen E., Sturmbauer C., Westneat M.W. (2002). Phylogeny of the Lake Tanganyika cichlid species flock and its relationship to the Central and East African Haplochromine cichlid fish faunas. Syst. Biol..

[36] Piálek L., Ríčan O., Casciotta J., Almirón A., Zrzavý J. (2012). Multilocus phylogeny of Crenicichla (Teleostei: Cichlidae), with biogeography of the *C. lacustris* group: Species flocks as a model for sympatric speciation in rivers. Mol. Phylogenetics Evol..

[37] Perazzo G.X., Noleto R.B., Vicari M.R., Gava A., Cestari M.M. (2013). Trends of karyotypical evolution in the pearl cichlid, *Geophagus brasiliensis*, from southern Brazil. Zoology.

[38] Getlekha N., Molina W.F., Cioffi M.B., Yano C.F., Maneechot N., Bertollo L.A.C., Supiwong W., Tanomtong A. (2016). Repetitive DNAs highlight the role of chromosomal fusions in the karyotype evolution of *Dascyllus* species (Pomacentridae, Perciformes). Genetica.

[39] Vicari M.R., Noleto R.B., Artoni R.F., Moreira-Filho O., Bertollo L.A.C. (2008). Comparative cytogenetics among species of the *Astyanax scabripinnis* complex: Evolutionary and biogeographical inferences. Genet. Mol. Biol..

[40] Greenwood P.H. (1974). The cichlid fishes of Lake Victoria, East Africa: The biology and evolution of a species flock. Bull. Br. Mus..

[41] Lecointre G., Améziane N., Boisselier M.C., Bonillo C., Busson F., Causse R., Chenuil A., Couloux A., Coutanceau J.P., Cruaud C. (2013). Is the species flock concept operational? The Antarctic shelf case. PLoS ONE.

[42] Varella H.R., Britzke R. (2016). *Apistogramma eleutheria* and *A. flavipedunculata*, two new species of dwarf cichlids from the rio Curuá on Serra do Cachimbo, Brazil (Teleostei: Cichlidae). Ichthyol. Explor. Freshw..

[43] Cioffi M.B., Kejnovskyý E., Marquioni V., Poltronieri J., Molina W.F., Diniz D., Bertollo L.A.C. (2012). The key role of repeated DNAs in sex chromosome evolution in two fish species with ZW sex chromosome system. Mol. Cytogenet..

[44] Lima-Filho P.A., Bertollo L.A.C., Cioffi M.B., Costa G.W.W.F., Molina W.F. (2014). Karyotype divergence and spreading of 5S rDNA sequences between genomes of two species: Darter and emerald gobies (*Ctenogobius*, Gobiidae). Cytogenet. Genome Res..

[45] Sember A., Bohlen J., Šlechtová V., Altmanová M., Symonová R., Ráb P. (2015). Karyotype differentiation in 19 species of river loach fishes (Nemacheilidae, Teleostei): Extensive variability associated with rDNA and heterochromatin distribution and its phylogenetic and ecological interpretation. BMC Evol. Biol..

[46] Supiwong W., Pinthong K., Seetapan K., Saenjundaeng P., Bertollo L.A.C., de Oliveira E.A., Yano C.F., Liehr T., Phimphan S., Tanomtong A. (2019). Karyotype diversity and evolutionary trends in the Asian swamp eel *Monopterus albus* (Synbranchiformes, Synbranchidae): A case of chromosomal speciation?. BMC Evol. Biol..

[47] Feldberg E., Bertollo L.A.C. (1985). Nucleolar organizing regions in some species of Neotropical cichlid fish (Pisces, Perciformes). Caryologia.

[48] Gornung E. (2013). Twenty years of physical mapping of major ribosomal RNA genes across the teleosts: A review of research. Cytogenet. Genome Res..

[49] Pires L.B., Giuliano-Caetano L., Dias A.L. (2010). Cytogenetic characterization of *Geophagus brasiliensis* and two species of *Gymnogeophagus* (Cichlidae: Geophaginae) from Guaiba Lake, RS, Brazil. Folia Biol..

[50] Froese R., Pauly D. (2018). FishBase. www.fishbase.org.

[51] Prieto J.L., McStay B. (2008). Pseudo-NORs: A novel model for studying nucleoli. Biochim. Biophys. Acta.

[52] Motta-Neto C.C., Marques A., Costa G.W.W.F., Cioffi M.B., Bertollo L.A.C., Soares R.X., Scortecci K.C., Artoni R.F., Molina W.F. (2018). Differential hypomethylation of the repetitive Tol2/Alu-rich sequences in the genome of *Bodianus* species (Labriformes, Labridae). Comp. Cytogenet..

[53] Robicheau B.M., Susko E., Harrigan A.M., Snyder M. (2017). Ribosomal RNA genes contribute to the formation of pseudogenes and junk DNA in the human genome. Genome Biol. Evol..

[54] Srivastava R., Srivastava R., Ahn S.H. (2016). The epigenetic pathways to ribosomal DNA silencing. Microbiol. Mol. Biol. Rev..

[55] Martins I.C., Portela-Castro A.L.B., Júlio Júnior H.F. (1995). Chromosome analysis of 5 species of the Cichlidae family (Pisces-Perciformes) from the Parana River. Cytologia.

[56] Mendonça M.N.C., Porto J.I.R., Feldberg E. (1999). Ocorrência de três citótipos em *Satanoperca* aff. *jurupari* (Perciformes, Cichlidae) no Catalão, Manaus, AM. Genet. Mol. Biol..

[57] Silva F.A., Carvalho N.D., Schneider C.H., Terencio M.L., Feldberg E., Gross M.C. (2016). Comparative cytotaxonomy of two species of fish from the genus *Satanoperca* reveals the presence of a B chromosome. Zebrafish.

[58] Molina W.F., Galetti P.M. (2002). Robertsonian rearrangements in the reef fish *Chromis* (Perciformes, Pomacentridae) involving chromosomes bearing 5S rRNA genes. Genet. Mol. Biol..

[59] Cioffi M.B., Martins C., Bertollo L.A.C. (2010). Chromosome spreading of associated transposable elements and ribosomal DNA in the fish *Erythrinus erythrinus*. Implications for genome change and karyoevolution in fish. BMC Evol. Biol..

[60] Oliveira E.A., Sember A., Bertollo L.A.C., Yano C.F., Ezaz T., Moreira Filho O., Hatanaka T., Trifonov V., Liehr T., Al-Rikabi A.B.H. (2018). Tracking the evolutionary pathway of sex chromosomes among fishes: Characterizing the unique XX/XY_1_Y_2_ system in *Hoplias malabaricus* (Teleostei, Characiformes). Chromosoma.

[61] Clark F.E., Conte M.A., Kocher T.D. (2018). Genomic characterization of a B chromosome in Lake Malawi cichlid fishes. Genes.

[62] Perazzo G.X., Noleto R.B., Vicari M.R., Gava A., Cestari M.M. (2018). B chromosome polymorphism in South American cichlid. Neotrop. Biodivers..

[63] Ready J.S., Sampaio I., Schneider H., Vinson C., Santos T., Turner G.F. (2006). Colour forms of Amazonian cichlid fish represent reproductively isolated species. J. Evol. Biol..

[64] Engelking B., Römer U., Beisenherz W. (2010). The intraspecific colour preference in mate choice by female *Apistogramma cacatuoides* Hoedeman, 1951 (Teleostei: Perciformes: Cichlidae). Vertebr. Zool..

[65] Seehausen O. (2006). Conservation: Losing biodiversity by reverse speciation. Curr. Biol..

[66] Price T.D., Bouvier M.M. (2002). The evolution of F1 postzygotic incompatibilities in birds. Evolution.

[67] Russell S.T. (2003). Evolution of intrinsic post-zygotic reproductive isolation in fish. Ann. Zool. Fenn..

[68] Molina W.F., Alves D.E.O., Araujo W.C., Martinez P.A., Silva M.F.M., Costa G.W.W.F. (2010). Performance of human immunostimulating agents in the improvement of fish cytogenetic preparations. Genet. Mol. Res..

[69] Gold J.R., Li Y.C., Shipley N.S., Powers P.K. (1990). Improved methods for working with fish chromosomes with a review of metaphase chromosome banding. J. Fish Biol..

[70] Sambrook J., Fritsch E.F., Maniatis T. (1989). Molecular Cloning: A Laboratory Manual.

[71] Howell W.M., Black D.A. (1980). Controlled silver-staining of nucleolus organizer regions with a protective colloidal developer: A 1-step method. Experientia.

[72] Sumner A.T. (1972). Simple technique for demonstrating centromeric heterochromatin. Exp. Cell Res..

[73] Pinkel D., Straume T., Gray J.W. (1986). Cytogenetic analysis using quantitative, high-sensitivity, fluorescence hybridization. Proc. Natl. Acad. Sci. USA.

[74] Pendás A.M., Moran P., Freije J.P., Garcia-Vazquez E. (1994). Chromosomal mapping and nucleotide sequence of two tandem repeats of Atlantic salmon 5S rDNA. Cytogenet. Cell Genet..

[75] White T.J., Bruns T., Lee S., Taylor J., Innis M.A., Gelfand D.H., Shinsky J.J., White T.J. (1990). Amplification and direct sequencing of fungal ribosomal RNA genes for phylogenetics. PCR Protocols: A Guide to Methods and Applications.

[76] Levan A., Fredga K., Sandberg A.A. (1964). Nomenclature for centromeric position on chromosomes. Hereditas.

